# Inhibition of West Nile Virus Multiplication in Cell Culture by Anti-Parkinsonian Drugs

**DOI:** 10.3389/fmicb.2016.00296

**Published:** 2016-03-07

**Authors:** Ana B. Blázquez, Miguel A. Martín-Acebes, Juan-Carlos Saiz

**Affiliations:** Department of Biotechnology, Instituto Nacional de Investigación y Tecnología Agraria y AlimentariaMadrid, Spain

**Keywords:** flavivirus, West Nile virus, neuroinvasive disease, Parkinson, antivirals, inhibition

## Abstract

West Nile virus (WNV) is a mosquito-borne flavivirus maintained in a transmission cycle between mosquitoes and birds, but it can also infect other vertebrates, including humans, in which it can cause neuroinvasive diseases. To date, no licensed vaccine or therapy for human use against this pathogen is yet available. A recent approach to search for new antiviral agent candidates is the assessment of long-used drugs commonly administered by clinicians to treat human disorders in drug antiviral development. In this regard, as patients with West Nile encephalitis frequently develop symptoms and features of parkinsonism, and cellular factors altered in parkinsonism, such as alpha-synuclein, have been shown to play a role on WNV infection, we have assessed the effect of four drugs (L-dopa, Selegiline, Isatin, and Amantadine), that are used as therapy for Parkinson’s disease in the inhibition of WNV multiplication. L-dopa, Isatin, and Amantadine treatments significantly reduced the production of infectious virus in all cell types tested, but only Amantadine reduced viral RNA levels. These results point to antiparkinsonian drugs as possible therapeutic candidates for the development of antiviral strategies against WNV infection.

## Introduction

West Nile virus (WNV) is a mosquito-borne flavivirus of the *Flaviviridae* family that belongs to the Japanese encephalitis (JEV) antigenic complex. This family includes other relevant human pathogens as JEV, Yellow Fever (YFV), Saint Louis encephalitis (SLEV), Dengue (DENV), and Zika (ZIKV) viruses, among others. WNV is maintained in an enzootic transmission cycle between mosquitoes and birds, but it can also infect other vertebrates, such as horses and humans (Martin-Acebes and Saiz, 2012). The virus is responsible for recurrent outbreaks of febrile illness and meningoencephalitis worldwide, accounting for hundreds of human deaths every year (Martin-Acebes and Saiz, 2012). Even though the great effort devoted in the past years to decipher the molecular biology of WNV and its interaction with the host immune system (Brinton, 2013; Suthar et al., 2013), no licensed vaccine or therapy for human use against this pathogen is yet available.

West Nile virus infection in humans is mainly subclinical, but approximately 20–40% of the infected people develop symptoms of disease that range from West Nile fever (fever, headache, lymphadenopathy, myalgia, fatigue, skin rash, diarrhea, and vomiting) to neurologic illness, and even to death (Donadieu et al., 2013). Neuroinvasive disease includes aseptic meningitis, encephalitis or an acute poliomyelitis-like syndrome (Davis et al., 2006; Sejvar, 2014). Patients with West Nile encephalitis frequently develop persistent movement disorders and tremors, and features of parkinsonism, including hypomimia, bradykinesia, and postural instability (Robinson et al., 2003). Likewise, cerebellar ataxia with associated truncal instability and gait disturbance, which induces symptoms similar to those of parkinsonism, has also been described in patients presenting WN encephalitis (Kanagarajan et al., 2003). Typically, these movement disorders resolve over time; however, tremor and parkinsonism may persist in patients recovering from WNV severe encephalitis (Sejvar, 2014). Moreover, it has been recently reported that parkinsonian features during WNV encephalitis are closely related to viral-induced cell death of dopaminergic neurons and the loss of dopamine signaling (Beatman et al., 2015). Besides WNV, different viral infections have been associated with parkinsonism (Jang et al., 2009), including those caused by other flaviviruses, such as JEV (Hamaue et al., 2006) or SLEV (Cerna et al., 1999). In fact, an early study reported that experimentally JEV-infected Fisher rats exhibited bradykinesia that was reversed by administration of L-dopa and of MAO (monoamine oxidase) inhibitors, suggesting that JEV infection induces Parkinson’s disease (PD) symptoms (Ogata et al., 1997). All these findings suggest that flavivirus infection could share cellular factors to those involved in parkinsonism.

Parkinson’s disease (PD) is a degenerative disorder of the central nervous system that mainly affects the motor system and was first described in detail in 1817 (Goetz, 2012). It is a consequence of a loss of dopamine-generating cells in the substantia nigra, a region of the midbrain. Early in the course of the disease, the most obvious symptoms are movement-related, including shaking, rigidity, slowness of movement, and difficulty with walking. The disease can be either primary, which is considered as idiopathic, or secondary, which can be caused, for instance, by toxins and viral infections. Nowadays, L-dopa is the most effective therapy available for treating the motor symptoms of PD, however, other medications (such as MAO B inhibitors, anticholinergics, amantadine, β-blockers, or dopamine agonist) are used in mild symptoms to avoid L-dopa-related motor complications (Connolly and Lang, 2014).

West Nile virus infections in humans provoke neurological disorders associated to the development of persistent movement disorders and tremors, similar to that observed in Parkinson’s disease patients. During this process, CNS neurons are affected, even leading to neuronal loss in the substantia nigra (Bosanko et al., 2003; Schafernak and Bigio, 2006), a process also observed in PD, in which a selective loss of dopamine-generating cells has been reported (Samii et al., 2004; Davie, 2008). In this regard, it has been recently described that neuronal expression of alpha-synuclein, a protein closely linked to PD (Stefanis, 2012), inhibits RNA viral infections in the central nervous system, suggesting that acute onset of parkinsonian features during WNV encephalitis could likely be due to viral-induced cell death of dopaminergic neurons that resulted in an acute loss of dopamine signaling (Beatman et al., 2015).

A recent approach to search for new antiviral agent candidates is the assessment of long-used drugs commonly administered by clinicians to treat human disorders, as part of drug repositioning (finding of new applications to licensed drugs). Among some of the drugs already tested as antivirals are lithium, statins, or valproic acid (Gilbert et al., 2005; Asenjo et al., 2008; Vazquez-Calvo et al., 2011). Likewise, the potential antiviral effect of multiple clinical compounds have also been revealed by massive screenings (Gastaminza et al., 2010). Since there is evidence supporting that WNV infection shares common points with Parkinson’s disease, we decide to study whether drugs used for the treatment of Parkinson’s disease could provide novel tools for antiviral intervention. In this way, we have assessed the effect of four antiparkinsonian drugs (L-dopa, Selegiline, Isatin, and Amantadine) in WNV multiplication in cultured cells from different origin. L-dopa, Isatin, and Amantadine treatments significantly reduced the production of infectious virus in all cell types tested, but only Amantadine reduced viral RNA levels. This results point to these drugs, especially Amantadine, as possible therapeutic candidates for the development of antiviral strategies against WNV infection.

## Materials and Methods

### Cells, Viruses, Infections, and Virus Titrations

Vero CCL81 (ATCC^®^ CCL-81^TM^), SH-SY5Y (ATCC^®^ CRL-2266^TM^), and HeLa (ATCC^®^ CCL-2^TM^) cells were grown (37°C, 5% CO_2_) in Eagle’s Minimum Essential Medium (EMEM; Lonza, Verviers, Belgium, cat n° BE12-125F) containing 5% fetal bovine serum (FBS; Hyclone, GE Healthcare, UK cat n° SV301360.03), in Dulbecco’s modified Eagle’s medium with nutrient mixture F-12 (DMEM/F-12(1:1); Gibco, Lifetechnologies, Carlsbad, CA, USA, cat n° 11330-032) containing 10% FBS, and in DMEM (Gibco, Lifetechnologies, cat n° BE12-614F) containing 10% FBS, respectively. All media were supplemented with 2 mM L-glutamine (Lonza, cat n° 17-905C) and penicillin-streptomycin (Lonza, cat n° DE17-602E).

All infectious virus manipulations were performed in our biosafety level 3 (BSL-3) facilities with a cell culture passaged New York/1999 (NY99) WNV strain [GenBank acc.: KC407666 (Cordoba et al., 2007; Martin-Acebes and Saiz, 2011)]. Virus infections were carried out as described (Martin-Acebes et al., 2014). Briefly, the viral inoculum was incubated with cell monolayers for 1 h at 37°C, and then removed before fresh medium containing fetal bovine serum was added. Viral titers were determined at 16 or 24 h post-infection (p.i.) by standard plaque assay in semisolid agarose medium. For this purpose, 10-fold serial dilutions of the supernatants in culture medium were added to subconfluent Vero cell monolayers grown on six-well tissue culture dishes. After 1 h of incubation at 37°C, viral inoculum was removed and fresh medium containing 2% fetal bovine serum, and 1% low-melting agarose (Conda, Cat. 8092.00) was added. Plates were incubated 72 h at 37°C and, then, fixed with 4% formaldehyde. Lysis plaques were visualized by staining with crystal violet. Usually, lysis plaques were scored in sample dilutions ranging from 10^–4^ to 10^–5^ of the initial sample, thus excluding possible effect of the remaining amounts of the drug with viral titration experiments. A multiplicity of infection (MOI) of 0.5 plaque forming units (PFUs)/cell were used in all experiments, except in those involving viral entry plaque assays (MOI of 1 PFU/cell), and immunofluorescence assays (MOI of 5 PFU/cell).

### Drug Treatments

The dopamine precursor L-dopa (cat n° PHR1271), the monoaminooxidase B inhibitors (MAOI B) Selegiline [R(-) deprenyl hydrochloride, cat n° M003] and Isatin (cat n° 114618), and Amantadine hydrochloride (cat n° A1260) were purchased from Sigma (St. Louis, MO, USA) and tested at different concentrations. Ammonium Chloride (NH_4_Cl, 25 mM, cat n° 168320) was from Merck (Darmstadt, Germany). In experiments with NH_4_Cl, extracellular pH was buffered with 25 mM HEPES at pH 7.5 (Sigma, cat n° H0887). Cells were infected, or mock infected, and drugs were added to the medium at different times prior to or after infection. Control cells were treated in parallel with the same amount of drug vehicle (cell culture media). Cell viability upon drug treatments was determined by measuring the cellular ATP content with CellTiter-Glo^®^ luminescent cell viability assay (Promega, Madison, WI, USA, cat n° G7571).

### Selectivity Index Determination

Selectivity Index value (SI) was determined as the ratio of cytotoxic concentration 50 (TC_50_) to inhibitory concentration 50 (IC_50_) for each treatment. To assess TC_50_, confluent SH-SY5Y cells in 96-well cell culture microplates were treated with a wide range of concentrations (from 0 to 300 mM), of each compound in triplicate. The treated cells were incubated 24 h at 37°C, and cell viability was determined with CellTiter-Glo^®^ luminescent cell viability assay according to the manufacturer protocol. Determination of IC_50_ was performed by titration of the PFU released to the supernatant of infected cultures treated with the same concentrations of the drugs.

### qRT-PCR

Viral RNA from culture supernatants was extracted using a commercial kit (Speedtools RNA virus extraction kit, Biotools B&M Labs S.A, Madrid, Spain cat n° 21142. The amount of viral RNA was determined by real-time fluorogenic reverse transcriptase PCR (RT-PCR) according to a previously published protocol (Lanciotti et al., 2000). The forward primer 5′-CAGACCACGCTACGGCG-3′, the reverse primer 5′- CTAGGGCCGCGTGGG-3′, and the probe 5′-FAM (6-carboxyfluorescein)- TCTGCGGAGAGTGCAGTCTGCGAT-3′-BHQ-1 (Black Hole Quencher-1), were used. Quantification was performed using High Scriptools-Quantimix Easy Probes Kit (Biotools) and a Rotor-Gene RG-3000 equipment (Corbett Research) as described (Merino-Ramos et al., 2015).

For quantification of cell-associated viral RNA, supernatants from infected cells were removed, cell monolayers were subjected to three freeze-thaw cycles and the RNA was extracted as described above. Extraction of cell-associated total RNA was performed using TRIzol reagent (Life Technologies, cat n° 15596-026). Total RNA was then treated with RQ1 DNase (Promega, Madison, WI, USA. cat n° M6101) to remove any contaminating DNA. cDNA was synthesized with Superscript III reverse transcriptase (Life technologies, cat n° 18080–18093) with oligo dT primer, and quantified by quantitative PCR (qPCR) with Sybr GreenER (Life technologies, cat n° 11762) using the internal control ribosomal RNA 18S specific primers (Biotools, cat n° 33007). Cell-associated viral RNA copies were calculated by normalizing to the internal control. The number of viral RNA copies is given as the number of genomic equivalents corresponding to the number of PFU/ml by comparison with the amount of RNA extracted from previously titrated samples (Blazquez and Saiz, 2010; Escribano-Romero et al., 2013).

### Antibodies and Staining

Mouse monoclonal antibody J2 against double-stranded RNA (dsRNA) was from English and Scientific Consulting (Scicon, Budapest, Hungary, cat n° T3605). Mouse monoclonal antibody 3.67G directed against WNV envelope protein was from Millipore (Temecula, CA, cat n° MAB8150), and Topro-3 (cat n° T3605) and secondary antibody against mouse IgGs coupled to Alexa Fluor-488 (cat n° 11001) were from Life Technologies.

### Immunofluorescence

Assays were carried out as described (Blazquez et al., 2013). Briefly, cells were grown on glass cover slips, fixed with 4% paraformaldehyde in PBS (15 min room temperature, rt), permeabilized with BPTG (1% BSA, 0.1% TritonX-100, 1 M glycine in PBS) for 15 min, incubated with primary antibody diluted in 1% BSA in PBS for 1 h, and then with fluorescently conjugated secondary antibody (45 min, rt). Finally, cells were incubated with Topro-3 (5 min, rt) and mounted with Fluoromount-G (SouthernBiotech, cat n° 0100-01). Between each step, samples were thoroughly washed with PBS. Samples were observed using a Leica TCS SPE confocal laser-scanning microscope, and the images were acquired using Leica Advanced Fluorescence Software, and processed using ImageJ (http://rsbweb.nih.gov/ij/) and Adobe Photoshop CS2.

### Statistical Analyses

Data are presented as mean ± SD. One-way analysis of variance (ANOVA) was performed using SPSS15 (SPSS Inc.). Differences were considered statistically significant at *P* < 0.05.

## Results

### Inhibition of WNV Multiplication in Neuronal Cells by Treatment with Antiparkinsonian Drugs

**FIGURE 1:**
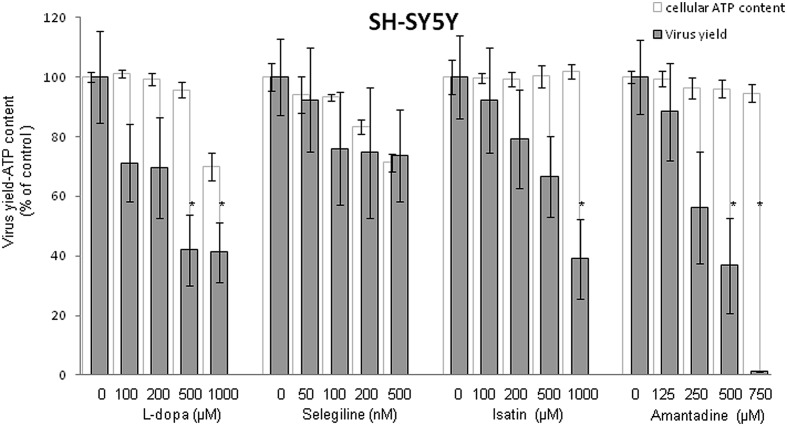
**Inhibition of WNV in SH-SY5Y cells treated with antiparkinsonian drugs and evaluation of the cytotoxicity of the treatments on SH-SY5Y cells by determination of cellular ATP content 24 h post-treatment.** Reduction of WNV infectious particle production and ATP measurement in cells treated with different concentrations of drugs. Cells were infected with WNV (MOI of 0.5 PFU/cell) and virus yield in culture supernatant was determined by plaque assay at 24 h p.i. Cell viability (estimated as ATP content) was determined in mock-infected cells treated with the same amount of drugs in parallel. Statistically significant differences are indicated: ^∗^*P* < 0.05.

Since neural tissues constitute a major target of WNV infection in human patients (Martin-Acebes and Saiz, 2012; Suen et al., 2014), the human neural cell line SH-SY5Y was chosen as a model to initially analyze the effects of antiparkinsonian drugs treatment on WNV infection. SH-SY5Y were infected with WNV and different concentrations of the drugs (L-dopa, Selegiline, Isatin, or Amantadine) were added 1 h p.i. to avoid their possible interference with virus entry. All drugs, except Selegiline, inhibited WNV multiplication in a dose dependent manner (**Figure 1**). The cytotoxicity of the treatments was analyzed in parallel by determination of the cellular ATP content (**Figure 1**), confirming that Isatin and Amantadine inhibited WNV multiplication with no significant toxicity up to 1000 μM and 750 μM, respectively. L-dopa inhibited WNV multiplication causing no detectable cytotoxicity up to 500 μM. Compounds that showed antiviral effects were then assessed to establish their SIs that determines the relative effectiveness of the drug in inhibiting viral replication compared to inducing cell death (**Table 1**). Overall, these results suggest that the antiparkinsonian L-dopa, Isatin, and Amantadine could constitute novel antiviral agents against WNV.

**Table 1:** Selectivity indexes (SIs) for antiparkinsonian drugs in SH-SY5Y cells.

	TC50 (mM)	IC50 (mM)	SI
L-dopa	70 ± 3.1	0.57 ± 0.09	122.81
Isatin	160 ± 4.6	0.78 ± 0.14	205.13
Amantadine	150 ± 1.4	0.37 ± 0.10	405.41

*Selectivity index was determined by quantifying the relation between cytotoxic concentration 50 (TC50) and inhibitory concentration 50 (IC50). TC50 is the concentration that results in the reduction of 50% of the ATP content of the host cells. IC50 is the concentration of drug at which virus production is inhibited by 50% determined by titration of culture supernatants standard plaque assay.*

### Effect of Antiparkinsonian Drugs on WNV Infection in Non-neural Cells

**FIGURE 2:**
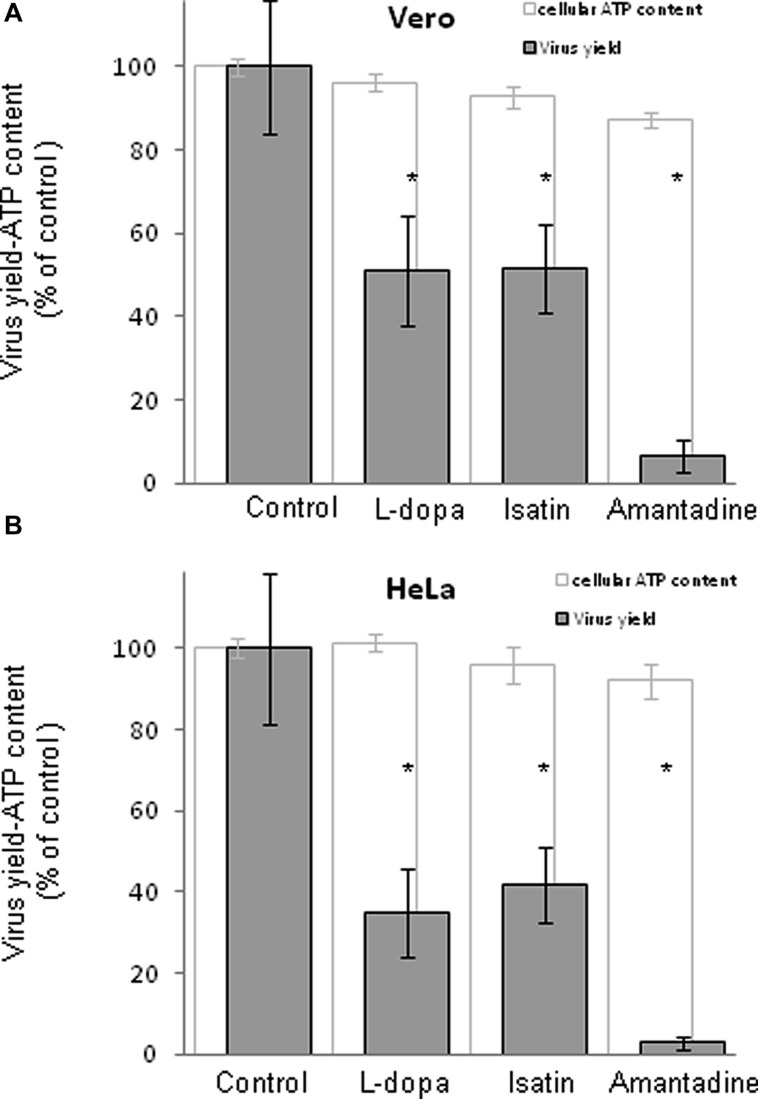
**Inhibition of WNV in cells treated with antiparkinsonian drugs, evaluation of the toxicity of the treatments on cells by determination of cellular ATP content 24 h post-treatment.** Drugs concentration were: L-dopa (500 μM), Isatin (1000 μM), and Amantadine (750 μM). **(A)** Reduction of WNV infectious particle production and ATP measurement in Vero cells treated with the drugs. Cells were infected with WNV (MOI of 0.5 PFU/cell) and virus yield in culture supernatant was determined by plaque assay at 24 h p.i. Cell viability (estimated as ATP content) was determined in mock-infected cells treated with the same amount of drugs in parallel. **(B)** Reduction of WNV infectious particle production and ATP measurement in HeLa cells. Cells were infected as in **(A)**. Statistically significant differences are indicated: ^∗^*P* < 0.05.

Since distribution of dopamine and MAO receptors are heterogeneously expressed not only in the central nervous system, but also in a wide range of cells, tissues, and organs (Missale et al., 1998; Pivonello et al., 2007), two non neural cell lines of different origin (Vero and HeLa) were also used to assess the antiviral effect of these drugs. These cell lines are widely used as models for the analysis of WNV infectious lifecycle (Krishnan et al., 2008; Gillespie et al., 2010; Martin-Acebes et al., 2014). Considering the results obtained in SH-SY5Y cells (**Figure 1**), the most effective concentration of drug that exerted minimal effects on cell viability (500, 1000, and 750 μM for L-dopa, Isatin, and Amantadine, respectively) was selected for the experiments in Vero and HeLa cells. Selegiline was not included in the analysis since this drug did not inhibit WNV infection in neural cells (**Figure 1**). WNV-infected Vero cells showed a statistically significant reduction in virus yield after treatment with L-dopa, Isatin, or Amantadine (**Figure 2A**). L-dopa and Isatin inhibited virus yield about 50%, while Amantadine did it by more than 90%. Higher inhibitory effects were even observed in HeLa cells upon treatment with all three drugs (**Figure 2B**). No significant effects on cell viability were observed at the concentrations of drugs used in any of the cell lines tested (**Figures 2A,B**). These results confirm the antiviral effect of L-dopa, Isatin, and Amantadine in WNV susceptible non-neural cells.

### Antiparkinsonian Drugs Did Not Affect Viral Entry into the Cell

**FIGURE 3:**
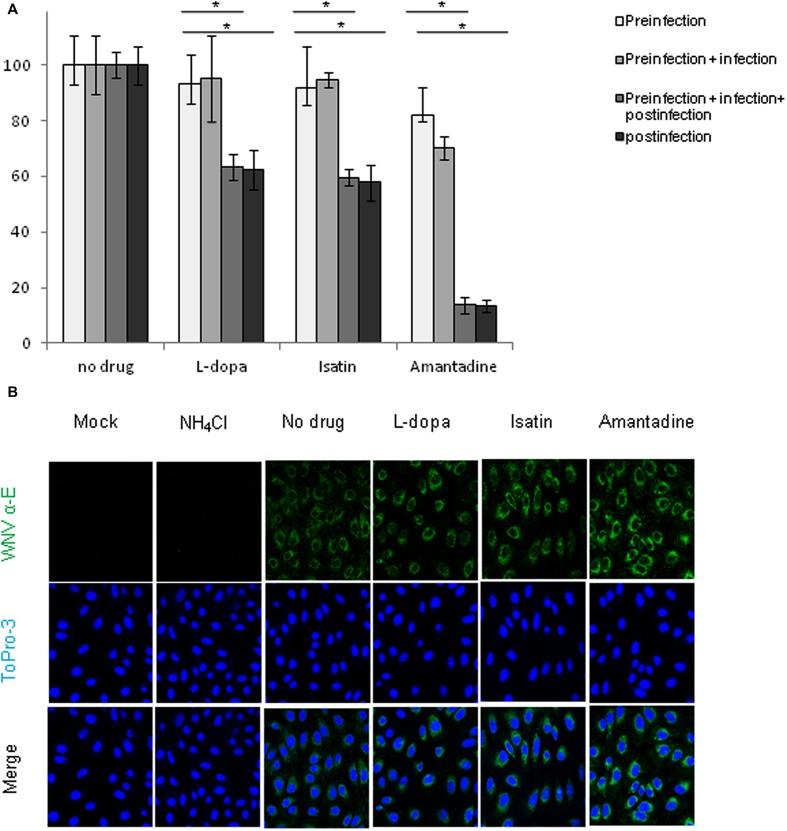
**(A)** Reduction of WNV infectious particle production in Vero cells treated with the drugs 1 h prior to infection and during the adsorption. Drugs concentration were: L-dopa (500 μM), Isatin (1000 μM), and Amantadine (750 μM). Supernatants were collected at 16 h p.i. **(B)** Visualization of the expression of WNV E glycoprotein in cells infected with WNV (MOI of 5 PFU/cell) and treated with drugs 1 h prior to infection and during the adsorption period. Drugs concentration were as in **(A)**. Vero cells were fixed at 16 h p.i. and processed for immunofluorescence using a monoclonal antibody against WNV-E protein and a secondary antibody coupled to Alexa Fluor 488. Nuclei were stained with ToPro-3. Mock-infected cells processed in parallel were included as control. WNV infected cells treated with NH_4_Cl 25 mM were also used as a positive control of a drug that inhibits viral entry. Statistically significant differences are indicated: ^∗^*P* < 0.05.

To assess the possible effect of the drugs during viral entry step, Vero cells were infected at high (MOI of 1 PFU/cell) to ensure that all cells were infected in a primary round of infection, thus avoiding possible secondary infections. Treatments were added to the culture medium following four different patterns: (a) 1 h previous to the infection and then removed; (b) 1 h pre-infection and during viral adsorption, when they were removed and fresh medium was added; (c) 1 h pre-infection and maintained throughout the rest of the assay (d) drugs were added only after 1 h of infection. The amount of infectious virus released into the culture medium was determined by plaque assay as early as 16 h p.i. WNV-infected Vero cells showed a statistically significant reduction in virus yield after treatment with L-dopa, Isatin, or Amantadine when the drugs were maintained after viral adsorption, but no significant effects were observed when used in pre-infection or adsorption steps (**Figure 3A**), confirming that none of the three compounds exerted any effect at the viral entry step. In addition, no significant differences on the extent of the inhibition between cells treated 1 h prior to infection and throughout the rest of the assay and those cells treated only from 1 h p.i., suggesting that the major inhibitory effect of the drug was produced after 1 h p.i.

To further verify these observations, the expression of WNV envelope (E) glycoprotein was detected by inmunofluorescence in WNV infected, or mock infected, Vero cells treated, or not, with the drugs. All the compounds were added 1 h prior to viral infection and kept only during the first hour of infection. After adsorption, treatments were removed and fresh medium was added to the cells. As a positive control for a compound that impairs WNV entry, ammonium chloride (25 mM NH_4_Cl) was added in the same conditions as the drugs. NH_4_Cl is a weak base that blocks organelle acidification and inhibits WNV infection at an entry step (Gollins and Porterfield, 1986; Zhang et al., 2007; Martin-Acebes et al., 2013). As expected, no expression of the WNV E glycoprotein was detected in mock infected or in WNV infected cells treated with NH_4_Cl (**Figure 3B**). Conversely, infected cells treated with L-dopa, Isatin, Amantadine, or non-treated cells revealed a high accumulation of WNV E glycoprotein. No statistically significant differences were observed in the amount of positive cells or in the intensity of the signal between treated and not treated cells. These results confirmed that the antiparkinsonian drugs here evaluated did not prevent viral entry.

### Amantadine Inhibits WNV Infection at a Replication Step

**FIGURE 4:**
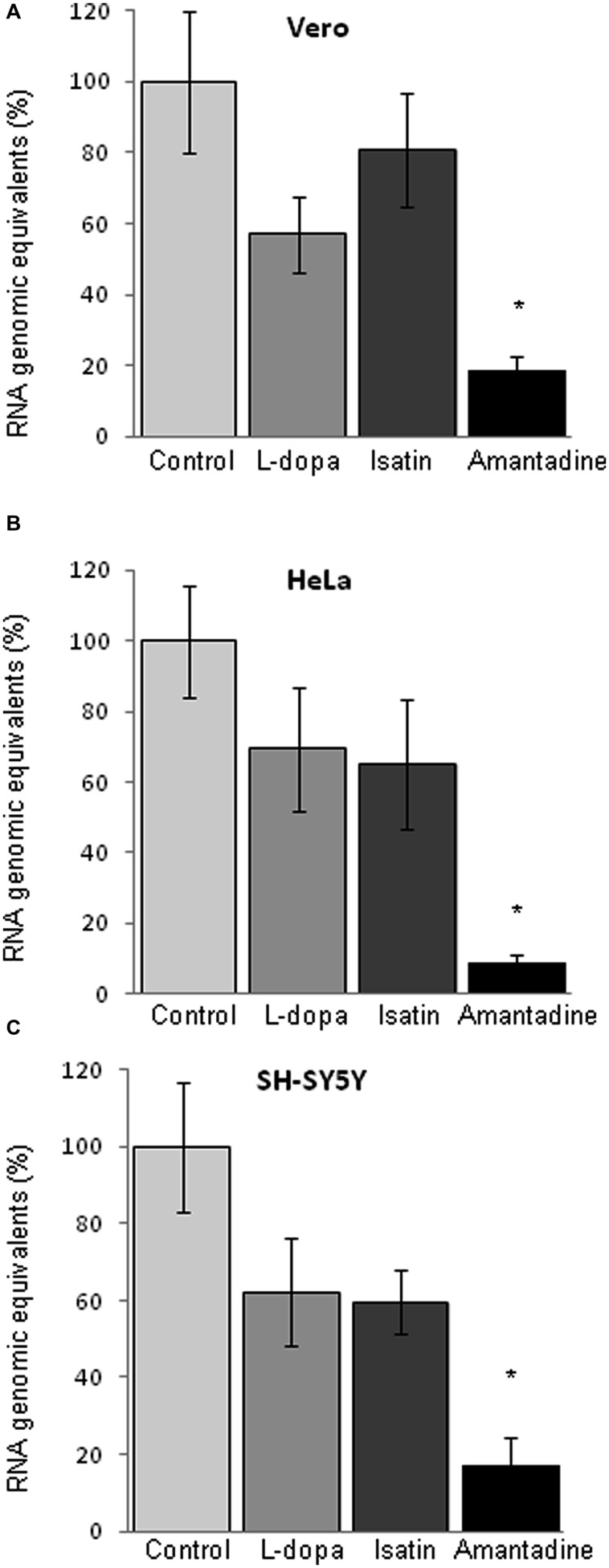
**Quantification of viral RNA in the culture supernatant of cells infected with WNV (MOI of 0.5 PFU/cell) by quantitative RT-PCR (24 h p.i.). (A)** Quantification in Vero **(A)**, HeLa **(B)**, and SH-SY5Y cells **(C)**. Drugs concentration were: L-dopa (500 μM), Isatin (1000 μM), and Amantadine (750 μM). Statistically significant differences are indicated: ^∗^*P* < 0.05.

The analysis of RNA released to the supernatant of infected cultures by quantitative RT-PCR showed a significant reduction on the amount of viral RNA released to the culture medium only by Amantadine (**Figures 4A–C**), contrary to what was observed after treatment with L-dopa or with Isatin, where no statistically significant inhibition of the release of genome containing units was recorded. Since these drugs also inhibited the production of infectious virus (**Figures 1** and **2**), these results suggest that they could be inducing the release of non-infectious genome-containing units.

**FIGURE 5:**
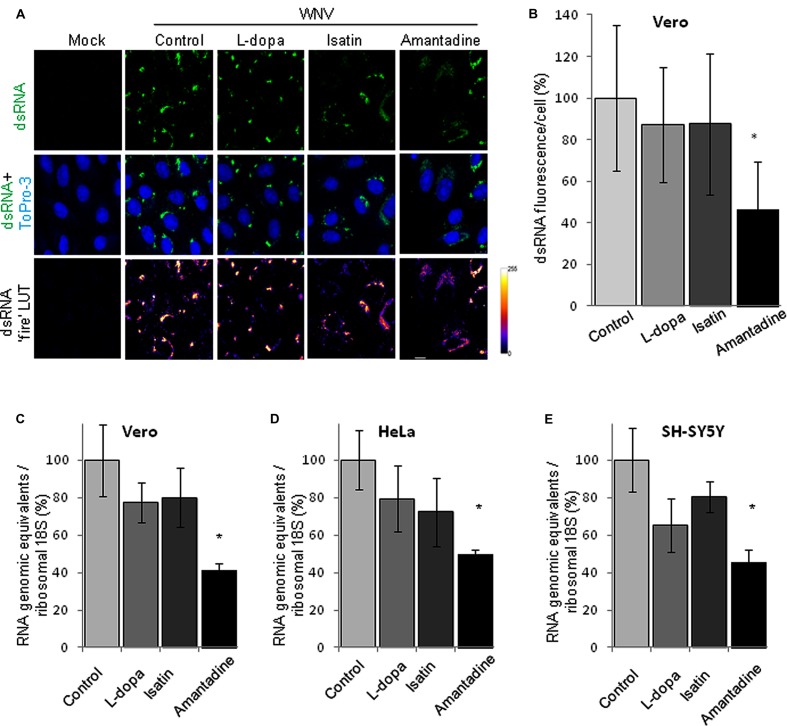
**(A)** Visualization of intracellular dsRNA accumulation in cells infected with WNV (MOI = 5 PFU/cell) and treated with drugs at 24 h p.i. Drugs concentration were: L-dopa (500 μM), Isatin (1000 μM), and Amantadine (750 μM). Vero cells were fixed and processed for immunofluorescence using monoclonal antibody against dsRNA J2 and a secondary antibody coupled to Alexa Fluor 488. Nuclei were stained with ToPro-3. Mock-infected cells processed in parallel are also included as a control. The panels in the last file display dsRNA staining with false coloring from dark purple to bright yellow by use of the fire lookup table (LUT) scheme to highlight differences in the intensities of the signals. Bars, 10 μm. **(B)** Quantification of the fluorescence intensity of dsRNA in cells infected and treated with drugs as shown in **(A)**. **(C–E)** Quantification of RNA genomic equivalents in cell-associated viral RNA in cell cultures infected with WNV (MOI of 0.5 PFU/cell) and treated with antiparkinsonian drugs determined by quantitative RT-PCR at 24 h p.i. Quantification of cell-associated WNV RNA in **(C)** Vero, **(D)** HeLa, and **(E)** SH-SY5Y cells. Genomic equivalents were normalized using cellular 18S RNA as an internal control. Statistically significant differences are indicated: ^∗^*P* < 0.05.

To further analyze the effects of these drugs on WNV genome replication, the amount of dsRNA intermediates, a well characterized maker of flavivirus replication complex, was analyzed by immunofluorescence in WNV infected Vero cells treated or not with the drugs. As expected, no dsRNA was detected in mock infected cells, whereas it was observed in infected cells (**Figure 5A**). The intensity of the signal in infected cells was statistically significant lower than that displayed by non-treated control infected cells only in the case of Amantadine (**Figure 5B**). Furthermore, quantification of cell-associated viral RNA by qRT-PCR confirmed the results obtained by immunofluorescence. Treatment with Amantadine showed a statistically significant reduction in the amount of cell-associated RNA relative to control samples and, to a lesser degree, this reduction was also observed with L-dopa or Isatin treatments, suggesting that treatment with either Amantadine, L-dopa, or Isatin, induce an impairment in WNV viral replication in cell culture (**Figures 5C–E**).

## Discussion

West Nile virus infections in humans have been reported to provoke meningitis and encephalitis associated to the development of persistent movement disorders and tremors. During this process, CNS neurons are affected, even leading to neuronal loss, gliosis, and neurofibrillary tangle formation in the substantia nigra (Bosanko et al., 2003; Schafernak and Bigio, 2006). In this regard, it is known that the selective loss of dopamine-generating cells in the substantia nigra is responsible for the development of Parkinson’s disease (Samii et al., 2004; Davie, 2008). Even more, it has been recently reported that neuronal expression of alpha-synuclein, a protein closely linked to PD (Stefanis, 2012), inhibited RNA viral infections in the central nervous system, suggesting that acute onset of parkinsonian features during WNV encephalitis are likely due to viral-induced cell death of dopaminergic neurons resulting in acute loss of dopamine signaling (Beatman et al., 2015).

Nowadays, the main used therapies for treating motor symptoms associated to parkinsonism are L-dopa (250 mg/day), dopamine agonists, and MAO-B inhibitors (selegiline 10 mg/day). Thus, following the recently trend to test drugs commonly administered to treat human disorders as innovative antiviral therapies, in this study, the effect on WNV multiplication of four different antiparkinsonian drugs has been addressed. A significant inhibition of WNV multiplication after treatment with L-dopa was observed in all the cell lines tested, independently of their neuronal origin or not. This probably reflects the fact that dopamine receptors are heterogeneously expressed in a wide range of cells, tissues and organs (Pivonello et al., 2007). Similar results were obtained with the MAO-B inhibitor (MAOI B) Isatin, which is not currently used in clinical therapy. In contrast, no positive results were recorded, independently of concentration or cell line used, when Selegiline, another MAOI B widely used in PD, was tested. These discrepancies, even though both drugs inhibit the same enzyme in the degradation of dopamine, could be due to their different mechanisms of action, being Isatin an endogenous inhibitor of the MAO B, while Selegiline belongs to the group of irreversible inhibitors of the enzyme. In fact, previous data have also reported a lower effect on bradykinesia and dopamine levels when Selegiline was administered to JEV-infected rats (Hamaue et al., 2004).

Among the antiparkinsonian drugs assayed, Amantadine showed the highest inhibitory effect in WNV infection, over 90% inhibition in virus yield in all cell lines tested. Amantadine began to be used in treating Parkinson’s disease in the 1960’s at dosages of 200 mg/day (Schwab et al., 1969) and, although its mechanism of action still remains unclear, it has been postulated to inhibit NMDA (*N*-methyl-D-Aspartate) receptors (Blanpied et al., 2005), and to release dopamine from the nerve endings of the brain cells together with the stimulation of a norepinephrine response DrugBank (n.d.). About the same time, Amantadine was recognized as an antiviral agent (Davies et al., 1964) that, since then, it has been used, among others, for influenza viral infections (Beigel and Bray, 2008; Razonable, 2011; Benschop et al., 2015). Our results indicate that Amantadine statistically significantly inhibited WNV replication, measured by either the release of viral RNA to the culture medium, or the quantification of cell-associated RNA, as well as the amount of dsRNA intermediates. The percentage of genome-containing units obtained after Amantadine treatment ranged, depending on the cell line, from 8 to 18% compared to untreated infected cells. The reduction in the amount of cell-associated viral RNA compared to untreated samples in any cell line tested and the lower amount of dsRNA intermediates observed in infected cells treated with this drug suggest an impairment of the proper development of WNV replication complexes. Differences in the WNV inhibition capability showed between L-dopa or Isatin treated cells when compared with that of Amantadine point that this drug is not only acting at dopamine receptor level, as different antiviral mechanisms of the drug have been proposed DrugBank (n.d.). For instance, Amantadine can inhibit virus multiplication at the maturation step by blocking viroporins participating in the adjustment of the pH of the environment in transport vesicle. However, to our knowledge, Hepatitis C virus (HCV) is the only member of the *Flaviviridae* family which contains viroporins in its genome (Madan and Bartenschlager, 2015). In fact, HCV viroporin protein p7-like is not present in WNV, and no other viroporins have currently been described for any members of the Flavivirus genus. Therefore, vioporins blocking does not seem to be involved in Amantadine inhibition of WNV multiplication.

On the other hand, the interference of Amantadine with virus uncoating has been previously described for influenza (Staničova et al., 2001). However, in our study the lack of effect of the tested drugs at the viral entry step has been demonstrated. No differences were observed by immunofluorescence in the expression of the viral E glycoprotein between treated and not treated cells when drugs were administered 1 h prior to infection, kept during viral adsorption, and then removed. Even more, our results indicated that virus yield was significantly decreased in infected Vero cells only when drugs were kept after infection, while it was not affected when the drugs were removed after viral adsorption.

The results presented in this study point to a possible therapeutic antiviral potential of antiparkinsonian drugs in the inhibition of neurotropic viruses such as WNV, which should be further confirmed *in vivo*. It has been thoroughly demonstrated that efficacy of antiviral drugs toward the cerebral viral load is often limited by the ability to cross the blood–brain barrier (BBB) (Groothuis and Levy, 1997; Strazielle and Ghersi-Egea, 2005). In this sense, the use of drugs widely applied in the treatment of neurological diseases overcomes the difficulties to reach the target, being a good starting point for antiviral development against WNV and other related neurovirulent flaviviral infections. In fact, it has to be considered that antiparkinsonian drugs can inhibit the parkinsonian symptoms of related flaviviruses in animal models (Hamaue et al., 2004). However, animal model experiments testing the dosages used in the patients treated with the antiparkinsonian drugs currently used in clinics that successfully inhibited WNV multiplication in cultured cells (L-dopa and amantadine), should be performed to evaluate if the *in vitro* effect observed against WNV could correlate with an *in vivo* effect.

## Author Contributions

AB has conducted the experiments and written the manuscript. MM-A and J-CS have contributed in the design of the experiments, and reading and correcting the manuscript.

## Conflict of Interest Statement

The authors declare that the research was conducted in the absence of any commercial or financial relationships that could be construed as a potential conflict of interest.
